# Metastatic Hypopharyngeal Carcinoma Involving the Stomach: A Case Report and Literature Review

**DOI:** 10.7759/cureus.102442

**Published:** 2026-01-27

**Authors:** Jad Kabbara, tannous Barakat, Mohamad Mouchli, Faris Shweikeh

**Affiliations:** 1 Anesthesiology, Lake Erie College of Osteopathic Medicine, Erie, USA; 2 Internal Medicine, Northeast Ohio Medical University, Rootstown, USA; 3 Gastroenterology, Fischer-Titus Medical Center, Westlake, USA; 4 Internal Medicine, Cleveland Clinic - Akron General, Akron, USA

**Keywords:** case report, endoscopy, gastric metastasis, head and neck cancer, hypopharyngeal squamous cell carcinoma, metastatic squamous cell carcinoma, molecular biomarkers, stomach metastasis

## Abstract

Hypopharyngeal squamous cell carcinoma (HSCC) is a highly aggressive malignancy constituting a small proportion of all head and neck cancers. While it has significant metastatic potential, spread to the stomach is exceedingly rare. A case of HSCC with metastasis to the stomach is presented, along with a review of the relevant literature.

A 71-year-old male patient with a history of HSCC was admitted to the hospital for melena and anemia. Prior to this presentation, he had documented metastases to the lungs and small bowel. Esophagogastroduodenoscopy (EGD) demonstrated a friable, non-bleeding gastric ulcer with raised margins. Biopsy findings were consistent with metastatic HSCC. He was considered for enrollment in potential oncological clinical trials.

We find that while metastasis to the stomach can occur in certain cancers such as breast, lung, and renal cell carcinoma, it is not common in hypopharyngeal cancer. A review of the literature reports only three previous cases. Multiple potential therapeutic molecular biomarkers have been identified for metastatic HSCC. The endoscopist should always consider metastatic disease when evaluating malignant stomach ulcers.

## Introduction

Metastatic cancer mostly occurs via spread through lymphatic drainage and blood supply. Some organs may be more common targets; however, metastases can develop at any site. Metastases to the stomach, specifically, are a fairly rare occurrence, with clinical incidence in autopsy series reporting a rate between 0.2% and 0.7% [1]. The main mechanism is not commonly understood and can be hematogenous, lymphatic, peritoneal dissemination, or direct invasion. The most common primary tumor sites of gastric metastases include breast cancer (27%), lung cancer (23%), and renal cell carcinoma (7%) [2].

Hypopharyngeal squamous cell carcinoma (HSCC) accounts for 6% of all head and neck cancers, with a worldwide incidence from 0.8 to 6 cases per 100,000 [3]. A highly aggressive malignancy, it is associated with increased age, male sex, tobacco abuse, alcohol use, and Caucasian race. While it has significant metastatic potential, spread to the stomach is very unusual, and here we present such a case. In keeping with our aims, we also review investigations on molecular mechanisms in HSCC metastasis and then outline recently reported cases from other primary cancers with gastric metastasis.

## Case presentation

A 71-year-old male patient with a history significant for HSCC was admitted to the hospital with melena. Three years ago, he had developed a chronic cough, an uncomfortable feeling in the throat, and swelling in the neck. Imaging revealed a left hypopharyngeal mass that was biopsied and showed invasive, moderately to poorly differentiated squamous cell carcinoma (SCC) that was p16-positive. He was treated with carboplatin, docetaxel, plus pembrolizumab, then transitioned to pembrolizumab alone. In the meantime, he received radiation to a right lung lesion due to progressive metastatic disease. His treatment was complicated by small bowel obstruction requiring resection. Pathology of two resected lesions in the jejunum revealed metastatic moderately differentiated SCC consistent with hypopharyngeal origin. He presented to our hospital with melanotic stool for six days. He was found to have anemia, and hemoglobin had dropped from 9.2 one month prior to 7.4, for which he was transfused two units of packed red blood cells.

**Figure 1:**
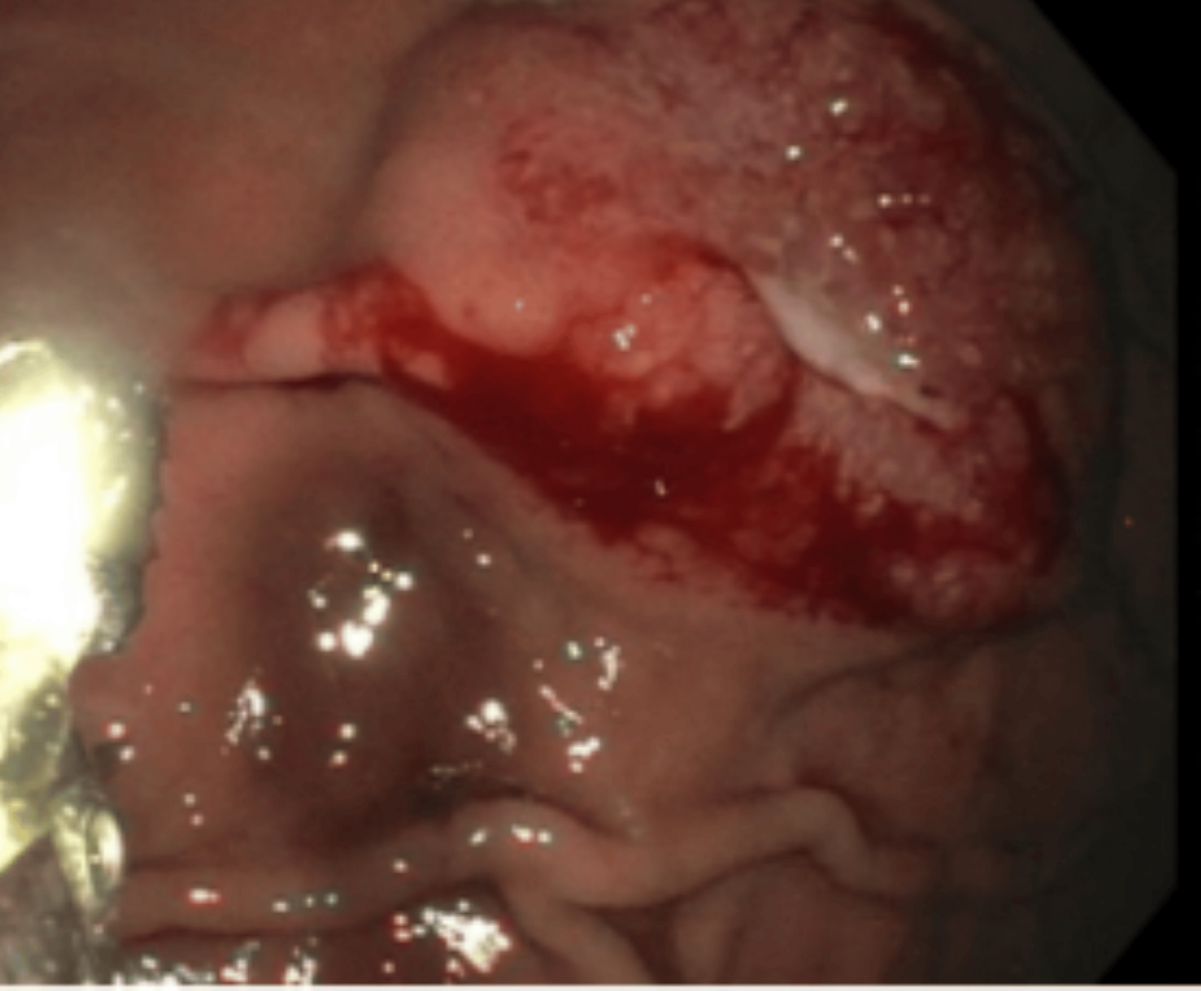
A friable ulcerated lesion with central crater and roll margins on the lesser curvature of the stomach, the mucosal surface is irregular and partially eroded. This endoscopic presentation is consistent with metastatic disease.

**Figure 2:**
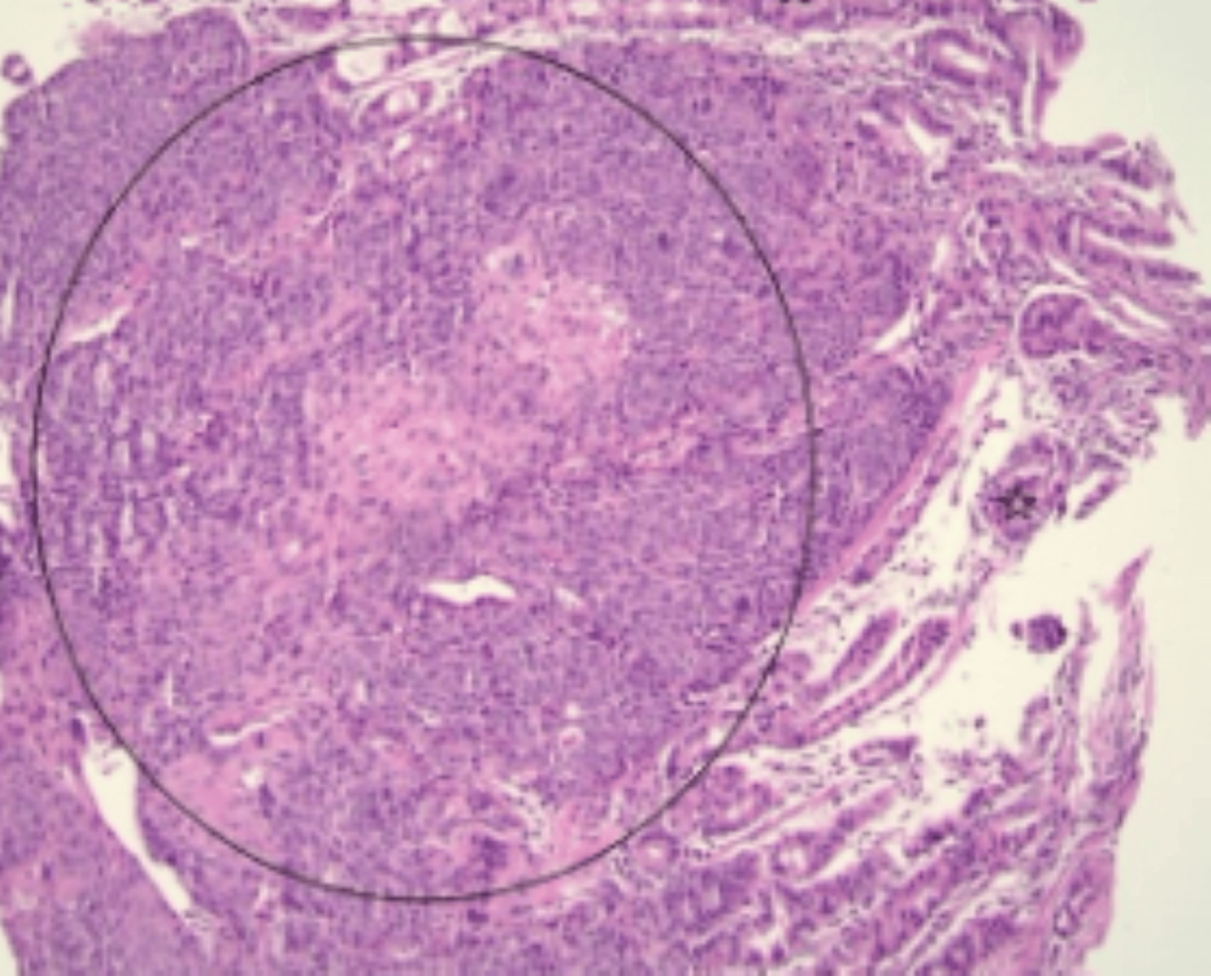
H&E-stained section at 20X, with the circle focus demonstrating cohesive nests of atypical squamous epithelial cells infiltrating the lamina propria beneath intact appearing gastric mucosa (stars). Tumor cells show eosinophilic cytoplasm and intracellular bridging consistent with metastatic SCC. H&E: Hematoxylin and eosin; SCC: Squamous cell carcinoma

Esophagogastroduodenoscopy (EGD) showed a friable, non-bleeding ulcer with central depression on the lesser curvature of the stomach with raised margins concerning for metastatic disease (Figure 1). Biopsies indicated metastatic HSCC in the stomach (Figure 2). The patient did not require endoscopic treatment since the ulcer was not bleeding. He followed up with oncology to determine eligibility for enrollment in clinical trials.

## Discussion

The case presented is distinctive when compared to the other three reported cases of HSCC metastasizing to the stomach. In contrast to our case, these patients either presented with a polypoid mass, a submucosal mass, or disease associated with percutaneous endoscopic gastrostomy (PEG) [4-6]. The case by Abdallah et al. was similar in clinical presentation but was hemodynamically unstable, requiring four units of packed red blood cells and admission to the ICU for close hemodynamic monitoring, highlighting the clinical variability of metastatic disease [6]. On CT imaging, multiple metastatic hepatic lesions and a gastric mass in the greater curvature were observed. The patient was treated with immunotherapy (pembrolizumab) and a palliative chemotherapy regimen [6]. Though also with stage IV hypopharyngeal cancer, this patient had a tracheostomy and PEG, which has its own mechanisms and risk for metastasis [7]. In the case by Wu et al., presentation occurred two years after cancer diagnosis; the initial symptoms two years prior were dysphagia and the presence of a bulging mass on the left upper neck that showed HSCC on biopsy. The patient then presented two years later with hematemesis, for which endoscopy showed two polypoid masses in the stomach and duodenum. The best form of treatment was surgery; however, the patient refused further advanced treatments, including surgery [4]. In the first reported case, published in 1985, a female patient presented with chest pain many months after HSCC treatment [5]. The gastric lesion in this case was a large submucosal mass, confirmed as SCC.

Due to the aggressiveness of HSCC and the higher propensity for late-stage disease at diagnosis, researchers have tried to elucidate molecular markers that are involved in metastasis of this malignancy. The basis for this is that malignant changes involve concordant, detectable changes at the level of gene expression. Moreover, scientific investigations in this realm can help elucidate clinical biomarkers that may aid in earlier identification and possible successful treatment of this often-deadly cancer. Recently, Zhou et al. revealed that the pyruvate kinase M2 (PKM2) gene was more highly expressed in those with lymph node metastasis than those without [8]. Further, patients with high PKM2 expression had a lower five-year survival on Kaplan-Meier analysis. Another study found that high expression of Stathmin1 (STMN1) in HSCC was directly associated with lymphatic vessel invasion [9]. Other investigators evaluated long noncoding RNAs (lncRNAs), such as lymphoid enhancer-binding factor 1 antisense RNA 1 (LEF1-AS1), and found that it supported metastatic spread of HSCC via a microRNA intermediate (miR-221-5p) [10]. Actin-binding proteins (ABPs) are theorized to regulate the actin cytoskeleton of cells, which in turn propels the process of metastasis, as investigated by Kakurina et al. [11]. Specifically, ABPs (e.g., RND3, CAP1, PFN1, etc.) can be involved in initiating metastatic processes such as epithelial-mesenchymal transition (EMT). Matrin3 (circMATR3), a type of circular RNA (circRNA), has also shown promise as a prognostic and therapeutic biomarker in HSCC due to its involvement in cellular proliferation, regulation of apoptosis, and invasion via multiple signaling cascades [12]. Finally, using reverse transcription-quantitative polymerase chain reaction (RT-qPCR) analysis and western blot, a study reported that HCLS1-associated protein X-1 (HAX-1) was overexpressed in HSCC, playing a significant role in metastasis [13]. All of these studies provide potential diagnostic and prognostic biomarkers for metastatic HSCC, with the central aim of improving overall survival.

On the other hand, a review of recent reports of metastatic disease to the stomach highlights the variability of this phenomenon. Breast cancers have previously been shown to exhibit gastric metastases. In a recent report, a patient presented 10 years after her initial cancer diagnosis with symptoms of upper abdominal discomfort and fatigue [14]. An EGD showed scattered polyps and mucosal infiltration involving the antrum, body, and cardia. Through immunohistochemical staining, it was determined that these lesions were intensely estrogen receptor (ER)-positive. The patient was started on endocrine therapy with palbociclib and tamoxifen, and she reached a stable disease course after one year. Others have also reported this tendency for multifocal spread of malignant breast cancer within the stomach [15,16]. Melanoma is notorious for metastasizing anywhere at any time, and this was highlighted in a recent case of an 85-year-old with a history of cutaneous melanoma of the right foot presenting with severe asthenia, severe anemia, and weight loss [17]. Endoscopy found four gastric lesions with central ulceration and blackish areas with a friable consistency, and immunohistochemistry confirmed primary cutaneous melanoma. Another report from a different cutaneous origin by Reise-Filteau et al. presented a 63-year-old female patient with a history of HIV and cutaneous SCC (cSCC) of the left temple [18]. The patient presented with functional decline, ascites, shortness of breath (SOB), and anemia. During the workup for her symptoms, CT scans of the chest showed multiple soft tissue lesions with a polypoid mass arising from the gastric antrum. EGD found a 2 × 2 cm submucosal ulcerated lesion in the gastric antrum and a 5 mm raised lesion in the duodenum, with genomic profiling linking these lesions to the primary cSCC. Palliative care was initiated for this patient due to the extent of her disease and poor prognosis, with her death occurring three weeks after admission.

Renal metastasis to the stomach was recently published by McIllwaine et al. in an 80-year-old female patient who previously underwent nephrectomy and presented with general malaise, weight loss, and acute hematemesis [19]. A 3 cm polypoid lesion on the greater curvature of the stomach was confirmed to be of renal origin. In a 2023 paper, there was a case of prostate cancer that began in a 65-year-old male patient with a prominent family history of cancer [20]. The patient presented nine years after his initial diagnosis of prostate cancer with epigastric discomfort, heartburn, decreased appetite, nausea, and anemia. An endoscopy was performed and found an ulcerated area on the greater curvature of the stomach. Through immunohistochemical staining, it was discovered that the tumor showed high prostate-specific antigen (PSA), indicating prostate metastasis. Radiation therapy was performed with 20 Gy in five fractions, but the patient ultimately declined further treatment and died after nine months of follow-up. Another uncommonly reported primary site is the thyroid, as published by Fuladi et al. [21]. A 72-year-old female patient with a primary diagnosis of anaplastic carcinoma of the left hemithyroid presented with persistent nausea and vomiting during adjuvant radiation therapy that was resistant to treatment with proton pump inhibitors (PPIs). Upper gastrointestinal endoscopy was performed and found seven-eight large polypoid lesions in the stomach (exact location not specified), with biopsy confirming origin from the primary anaplastic thyroid carcinoma. The patient died 2.5 months after presentation due to hemorrhagic shock.

Treatment options for metastatic HSCC are driven by systemic immunotherapy, with the current first line being either pembrolizumab therapy alone or combined with platinum-based chemotherapy, which has demonstrated overall survival benefits compared with the previous chemotherapeutic regimen [22]. Conventional second-line options include taxane-based regimens or cetuximab-containing combinations in patients who have progressed after exposure to platinum-based chemotherapy [23]. Control of tumor-related bleeding can be achieved through endoscopic hemostasis, and short-course radiotherapy is an effective option for local control when bleeding or pain persists [24]. Table 1 summarizes these cases, along with others.

**Table 1:** Summary of findings in reported cases with metastatic HSCC to the stomach along with others from other origins published recently HSCC: Hypopharyngeal squamous cell carcinoma; cSCC: Cutaneous squamous cell carcinoma; SOB: Shortness of breath; NR: Not reported; ER: Estrogen receptor

Study	Age/Sex	Primary site	Presentation	Endoscopy findings	Location (in stomach)	Treatment	Outcome	Special Aspects
Abdalla et al. [6]	62/M	Hypopharynx	Black-colored stool, epigastric tenderness	Large, ulcerated, non-circumferential mass	Greater curvature	Immunotherapy, palliative chemotherapy	Stabilized with oncology follow-up	NA
Moshref et al. [20]	65/M	Prostate	Epigastric pain, heartburn, decreased appetite, nausea, worsening anemia	Ulcerated area	Greater curvature	20 GY radiation in 5 fractions, declined further therapy	Death at 9 months	NA
Paricio et al. [17]	85/F	Cutaneous melanoma of right foot	Severe asthenia, anemia, weight loss	4 gastric lesions with central ulceration and blackish area and friable consistency	Body and subcardial region	NR	NR	Positive for S100, HMB-45, Melan-A
McIlwaine et al. [19]	80/F	Kidney (clear cell)	Malaise, weight loss, raised inflammatory markers, acute hematemesis	Polypoidal lesion, irregular pit pattern	Greater curvature	None, patient preference	NR	Pulmonary metastases also
Fu et al. [14]	63/F	Lobular carcinoma of breast	Upper abdominal discomfort and fatigue	Scattered polyps and mucosal infiltration throughout the stomach	Gastric antrum, body, cardia	Endocrine therapy	Reached stable disease course	Immunohistochemistry: ER positive
Reise-Filteau et al. [18]	63/F	cSCC of right temple, HIV+	Functional decline, ascites, SOB, anemia	Submucosal ulcerated lesion, raised lesion on duodenum	Gastric antrum	Palliation	Death 3 weeks after readmission	Genomic profiling: MetS had similar mutations as cSCC,
Fuladi et al. [21]	72/F	Thyroid (anaplastic carcinoma)	Nausea, vomiting	7-8 large polypoidal lesions	NR	NR	Death due to hemorrhagic shock following admission	NA
Wu et al. [4]	68/M	Hypopharynx	Hematemesis	Polypoid mass with a ragged surface and irregular ulcerations	Greater curvature	None, patient died, refused invasive surgical treatment	Death due to aspiration pneumonia and multiorgan failure	Metastasized to duodenum as well
Glick et al. [5]	49/F	Hypopharynx	Continuous chest pain	Submucosal mass, lesser curvature	Gastric cardia	NR	NR	NA

## Conclusions

Though metastasis to the stomach can happen in certain cancers, hypopharyngeal cancer does not commonly do so. Review of the literature reveals very few such cases. The case compares and contrasts in various ways with those previously reported, adding significant findings to our current knowledge. Multiple molecular biomarkers have been reported in the pathogenesis of metastatic HSCC, including novel RNAs such as lncRNAs, circRNA, and miRNA. These can serve as future oncological targets with the hope of improving patient outcomes. Current management should focus on treating the underlying disease, which generally entails systemic chemotherapy. Finally, the endoscopist should consider metastatic disease when evaluating malignant stomach ulcers.
